# Nonmosaic Isodicentric Y Chromosome: A Rare Cause of Azoospermia— From Genetics to Clinical Practice

**DOI:** 10.1155/2020/8828740

**Published:** 2020-07-25

**Authors:** Jolijn Van Cauwenberghe, Sigri Beckers, Peter Coremans

**Affiliations:** ^1^Antwerp University Hospital, Department of Diabetology-Endocrinology, Wilrijkstraat 10, 2650 Edegem, Belgium; ^2^Antwerp University Hospital, Department of Genetics, Wilrijkstraat 10, 2560 Edegem, Belgium; ^3^AZ Nikolaas, Department of Diabetology-Endocrinology, Moerlandstraat 1, 9100 Sint-Niklaas, Belgium

## Abstract

Azoospermia is diagnosed when no spermatozoa can be detected after centrifugation of seminal fluid on at least two separate occasions. A number of genetic disorders can be related to nonobstructive azoospermia, and in up to 15% of azoospermic males, a genetic disorder is diagnosed. A 36-year-old male with nonobstructive azoospermia was referred to our department of diabetes and endocrinology due to an aberrant testicular biopsy. The biopsy showed a disrupted spermatogenesis with a maturation arrest at the spermatocyte level in most tubuli seminiferi while others showed a Sertoli cell-only syndrome. Screening for Y chromosome microdeletions on peripheral blood using molecular analysis detected a terminal deletion of AZFbc. The result of karyotyping and fluorescence in situ hybridization (FISH) described an isodicentric Y chromosome with karyotype 46,X,idic(Y)(q11.22). Based on this case and the current available literature, we conclude that performing a testicular biopsy in patients with a nonmosaic idic(Y)(q) is not meaningful and that the prognosis on infertility is poor. Biological fatherhood is extremely unlikely in these patients, and proper counselling should be provided.

## 1. Introduction

Ten to 15% of males with infertility are found to have a complete absence of spermatozoa in the ejaculate, called azoospermia [1]. Azoospermia is diagnosed when no spermatozoa can be detected after centrifugation of seminal fluid on at least two separate occasions. Azoospermia can be caused by endocrine abnormalities, intrinsic spermatogenesis disorders, or posttesticular causes [2].

A genetic cause can be found in up to 15% of males with azoospermia [3]. Sex chromosomal abnormalities, diagnosed through karyotyping, are one of the most common genetic abnormalities and include numerical and structural abnormalities. Two-thirds of chromosomal abnormalities in infertile men are caused by the well-known numerical disorder Klinefelter syndrome (47,XXY) [4, 5]. Furthermore, microdeletions of the azoospermia factor region (*AZF* gene) on the Y chromosome are another important type of genetic aberration that can be found in approximately 10% of nonobstructive azoospermic males [6]. These microdeletions are divided into deletions of AZFa, AZFb, and AZFc or combined deletions and can be detected using polymerase chain reaction (PCR) techniques. The clinical consequences are determined by the site and length of the deletion [6].

Isochromosome Y (iso(Y)) and isodicentric chromosome Y (idic(Y)) are structural Y chromosome aberrations that are associated with male infertility and nonobstructive azoospermia [6–10]. An idic(Y) is formed due to an aberrant homologous crossing over between opposite arms of a palindrome during spermatogenesis. This can result in a duplication of the short arm and proximal long arm and a deletion of a part of the long arm and is referred to as idic(Y)(q). Chromosomes affected in this way contain two centromeres and are often unstable during mitosis. Therefore, most cases described are mosaics with a 45,X cell line [7, 10–12]. A broad range of phenotypes are reported in these cases, ranging from male to abnormal female or individual with ambiguous genitalia [13]. Knowledge about this specific chromosomal aberration is important considering the great impact on the possibility of assisted reproductive technology (ART) [1, 14].

We describe a patient with nonmosaic isodicentric Y chromosome (46,X,idic(Y)(q11.22)) presenting with nonobstructive azoospermia. Informed consent was obtained.

## 2. Case Report

**Figure 1:**
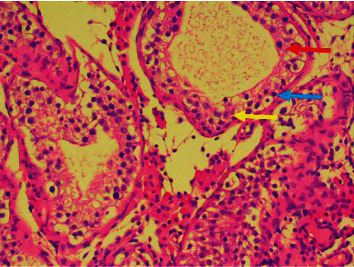
Testicular biopsy with maturation arrest at the spermatocyte level. Red arrow = spermatocyte. Blue arrow = spermatogona. Yellow arrow = Sertoli cell.

**Figure 2:**
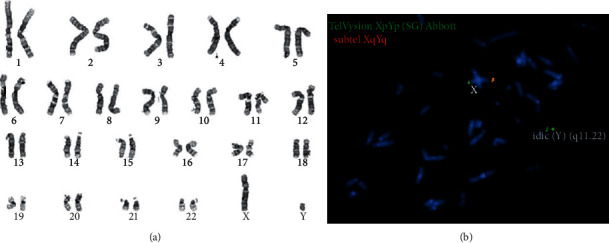
Karyotyping and fluorescence in situ hybridization (FISH) analyses. (a) Conventional chromosome analysis of cultured peripheral lymphocytes identified an idic(Y)(q11.22) chromosome. (b) FISH analysis was performed using probes for the telomeric regions of both the X and Y chromosomes. Green signals indicate the pter-region of the X and Y chromosomes (Abbott TelVysion Xp/Yp probe). Red signals indicate the qter-region of the X and Y chromosomes (in-house subtelomeric XqYq probe). FISH analysis demonstrated that the aberrant Y chromosome contained 2 signals for the Yp subtelomeric probe and absence of the Yq subtelomeric probe. This confirmed the presence of an idic(Y)(q).

A 36-year-old male was referred because of an aberrant testicular biopsy. The biopsy showed disrupted spermatogenesis with maturation arrest at the spermatocyte level in most tubuli seminiferi (Figure 1), while others showed a Sertoli cell-only syndrome. The referring urologist performed the biopsy in the context of primary infertility with confirmed azoospermia on the semen sample. There was no history of cryptorchidism, mumps, orchitis, testicular cancer, radiation, surgery or testicular trauma, nor a family history of infertility. The only medication our patient took was levothyroxine for hypothyroidism. There was no history of drug use or abuse. On physical examination, we found a man of normal constitution with normal virilization. Scrotal palpation detected the presence of bilateral vas deferens and small testicular volumes of 6 to 8 millilitres using an orchidometer. Ultrasonography of both testes confirmed the small testis (2 × 1.4 × 3.7 cm left and 2.2 × 1.4 × 3.4 cm right), and there were no suspicious lesions to withhold. Blood tests showed an elevated follicle-stimulating hormone (14.50 U/l, normal value: 0.7–10.8 U/l) with a normal luteinizing hormone (5.0 U/l, normal value 2.0–5.3 mU/l) and low normal testosterone (302.0 ng/dl, normal value 300–1000 ng/dl). Prolactin levels were normal (7.7 *μ*g/L, normal value 1.6–18.8 *μ*g/L). Screening for Y chromosome microdeletions on peripheral blood using molecular analysis detected a terminal deletion of AZFbc. The result of karyotyping and fluorescence in situ hybridization (FISH) on cultured peripheral lymphocytes described an isodicentric Y chromosome with karyotype 46,X,idic(Y)(q11.22) (Figure 2). These results implicate the impossibility of biological fatherhood for this patient, and we, therefore, referred our patient and the partner to a multidisciplinary fertility clinic for further counselling.

## 3. Discussion

**Figure 3:**
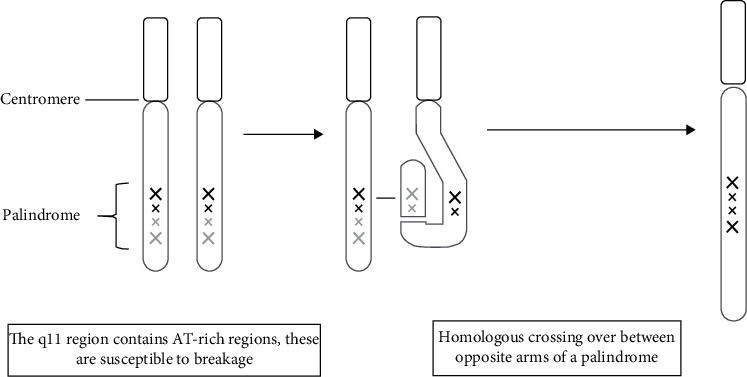
Schematic representation of aberrant homologous crossing over between opposite arms of a palindrome during spermatogenesis with the formation of an isodicentric Y chromosome.

**Figure 4:**
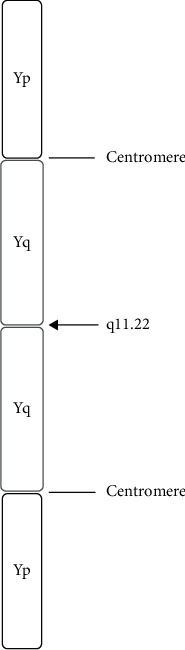
Schematic representation of isodicentric Y chromosome idic(Y)(q11.22). The breakpoint (arrow) is located at q11.22 (long arm), with a duplication of short arm, centromere, and proximal long arm and loss of all distal long arm material.

In azoospermic males, genetic testing is strongly advised considering that a genetic disorder can be diagnosed in up to 15 percent of the cases [14]. To ensure proper counselling for patients confronted with infertility, a correct genetic diagnosis is essential. In addition, expensive and invasive diagnostic tests and treatments can be avoided. This case describes a rare genetic disorder associated with nonobstructive azoospermia: the isodicentric Y chromosome. Although data are lacking for the general population, in infertile men with an abnormal Y chromosome, idic(Y) occurs in 15–30% [15]. An idic(Y) is formed due to an aberrant homologous crossing over between opposite arms of a palindrome during spermatogenesis. This can result in a duplication of the short arm and proximal long arm and a deletion of a part of the long arm and is referred to as idic(Y)(q). The q11 region of the long arm of the Y chromosome is susceptible as a breakpoint for this aberrant crossing over due to the presence of AT-rich regions which are fragile sites for breaking. In addition, the q11.2 region contains a direct repeat [16] (Figures 3 and 4).

The vast majority of reported idic(Y)(q) cases (55–100%) present as mosaics with a 45,X cell line due to the instability during mitosis [7, 8, 10–12]. This mosaicism provides a broad range of phenotypes, from phenotypically normal males to even phenotypical females [12]. There is a known variability in the distribution of idic(Y) in different tissues, with higher variability in gonadal tissue. The sex differentiation is believed to be depending on this mosaicism in gonadal tissue [16].

The case we describe showed a 46,X,idic(Y)(q11.22) karyotype without mosaicism, and in contrast to most published cases, we performed a complete work-up (physical examination, hormonal evaluation, and testis biopsy) (Figure 3.) We made a comparison with 12 published cases of infertile men with nonmosaic idic(Y)(q) in order to provide guidance for clinical practitioners confronted with similar patients (Table 1). The majority of the patients had small testes, and all but one case had azoospermia on a repeated semen sample. The one case, described by Antonelli et al., showed spermatocytes on the cytomorphological sediment in the sperm sample, which cannot be used in ART. To the best of our knowledge, testicular histology was reported for only six cases of infertile men with nonmosaic idic(Y)(q). All these cases showed a severe disruption of spermatogenesis with maturation arrest and/or Sertoli cell-only histology. In the diagnostic evaluation of the azoospermic males, current guidelines state that a diagnostic testicular biopsy is indicated in men with normal testicular size and normal reproductive hormones in order to distinguish between obstructive and a nonobstructive azoospermia. In azoospermia with high FSH, a testicular biopsy can determine the presence of a normal or abnormal spermatogenesis, although it does not provide accurate prognostic information [14, 17]. Since the genetic testing provides a clear diagnosis of nonobstructive azoospermia, a testicular biopsy in patients with idic(Y)(q) is not meaningful. This invasive technique should, therefore, not be performed before genetic testing is completed in patients with nonobstructive azoospermia.

Furthermore, both Mascarenhas et al. and Takeda et al. performed a testicular sperm extraction (TESE) and micro-TESE, respectively, in a nonmosaic idic(Y)(q) male without success, meaning there were no spermatozoa retrieved [6, 7]. All these data show the poor prognosis of fertility in patients with nonmosaic idic(Y)(q). As there were no spermatozoa on testicular biopsy and micro-TESE in the described cases up till now, the use of ART seems limited, and biological fatherhood is extremely unlikely in patients with a nonmosaic idic(Y)(q).

In infertile men with mosaic idic(Y)(q), the prognosis for infertility is less clear. Overall, all infertile men with idic(Y)(q) present with a nonobstructive azoospermia [8–10]. Nevertheless, one of the mosaic idic(Y)(q) cases reported by Antonelli et al. showed a low number of spermatocytes on the semen sample (<0.1 10^6^/ml). [10] Furthermore, Kim et al. report a normal spermatogenesis on testicular biopsy in an azoospermic male with mosaic idic(Y)(q) [8]. Thus, despite the low chance of success, ART cannot be completely excluded in infertile men with mosaic idic(Y)(q).

## 4. Conclusions

To the best of our knowledge, this is only the second case of a nonmosaic idic(Y)(q) that had an extensive evaluation. Based on our case report and the available literature, we conclude that performing a testicular biopsy in patients with a nonmosaic idic(Y)(q) is futile. Furthermore, the prognosis on infertility is poor. It seems that retrieval of spermatozoa is impossible, and therefore, biological fatherhood is extremely unlikely in these patients. Proper counselling for patients and their partners should be provided.

## Figures and Tables

**Table 1:** Summary of idic(Y)(q) cases described in the literature.

	idic(Y)	Testicular volume (ml)	Semen	Hormone levels			Testicular biopsy	AZF microdeletion	TESE
				FSH	LH	Testosterone			
Current case	46,X,idic(Y)(q11.22)	8	AZ	Elevated	Normal	Normal	MA + SCO	AZF bc	

Kalantari et al. [9]	46,X,idic(Y)(q11.22)	—	AZ	—	—	—	MA + SCO	AZF bc	
	46,X,idic(Y)(q11.22)	—	AZ	—	—	—	MA + SCO	AZF bc	
	46,X,idic(Y)(q11.22)	—	AZ	—	—	—	MA + SCO	AZF bc	

Kim et al. [8]	46,X,idic(Y)(q11.2)	11	AZ	Normal	Normal	Normal	MA	AZF bc	
	46,X,idic(Y)(q11.21)	5	AZ	Low	Low	Normal	—	AZF abc	
	46,X,idic(Y)(q11.222)	12	AZ	Normal	Normal	Normal	—	AZF bc	
	46,X,idic(Y)(q11.2)	2	AZ	Normal	Normal	Normal	—	AZF abc	
	46,X,idic(Y)(q12)	3	AZ	Normal	—	Normal	MA	Normal	

Antonelli et al. [10]	46,X,idic(Y)(q11.2)	Normal^1^	AZ	Normal	Normal	Normal	—	AZF bc	
	46,X,idic(Y)(q12)	Normal^1^	Spermatocytes	Normal	Normal	Normal	—	Normal	

Takeda et al. [7]	46,X,idic(Y)(q11.2)	7	—	Elevated	Normal	Normal	SCO	—	No spermatozoa^2^

Mascarenhas et al. [6]	46,X,idic(Y)(q11.21)	—	AZ	Elevated	—	—	—	—	No spermatozoa

AZ = azoospermia; MA = maturation arrest; SCO = Sertoli cell-only; TESE = testicular sperm extraction. ^1^No detailed testicular volume reported; ^2^micro-TES.
